# Increased levels of serum IL-17 and induced sputum neutrophil percentage are associated with severe early-onset asthma in adults

**DOI:** 10.1186/s13223-021-00568-9

**Published:** 2021-07-05

**Authors:** Dandan Chen, Yu Zhang, Can Yao, Binbin Li, Sinian Li, Wenwen Liu, Rongchang Chen, Fei Shi

**Affiliations:** 1 Key Laboratory of Shenzhen Respiratory Diseases, Institute of Shenzhen Respiratory Diseases, Shenzhen People’s Hospital (The First Affiliated Hospital of Southern University of Science and Technology, The Second Clinical Medical College of Jinan University), 1017 Dongmen North Road, Shenzhen, 518020 Guangdong China; 2 Emergency Department, Key Laboratory of Shenzhen Respiratory Diseases, Institute of Shenzhen Respiratory Diseases, Shenzhen People’s Hospital (The First Affiliated Hospital of Southern University of Science and Technology, The Second Clinical Medical College of Jinan University), 1017 Dongmen North Road, Shenzhen, 518020 Guangdong China

**Keywords:** Early-onset, Severe asthma, IL-17, Neutrophil

## Abstract

**Background:**

Differences between adult patients with severe early-onset and late-onset asthma have not been well studied.

**Objectives:**

To determine the phenotypic distinction regarding age at onset in patients with severe asthma.

**Methods:**

The present study enrolled thirty-two patients with severe early-onset (onset age < 12 years) asthma and thirty-two patients with severe late-onset (onset age > 12 years) asthma. Severe asthma was defined according to Global Initiative for Asthma criteria. The clinical, spirometric, and laboratory parameters were collected for group comparisons.

**Results:**

Among the 64 patients included (mean age, 46.22 ± 13.90 years; 53.1% male), the mean percent of predicted forced expiratory volume in 1 s (FEV1) was 68.43 ± 20.55%. Patients with severe early-onset asthma had a younger age, longer duration of asthma, higher rate of family history, and better small-airway function (MEF25% and MMEF75/25%) compared with severe late-onset asthma. Furthermore, levels of serum IL-17 and sputum neutrophil percentage were significantly higher for patients with severe early-onset asthma (*P* = 0.016, 0.033, respectively). Multiple logistic regression analysis revealed that increased serum IL-17 (odds ratio = 1.065, *P* = 0.016) was independently associated with severe early-onset asthma. The combination of serum IL-17 and sputum neutrophil percentage yielded a sensitivity of 80.0% and a specificity of 86.7% for identifying patients with severe early-onset asthma.

**Conclusions:**

Patients with severe early-onset asthma exhibit elevated levels of serum IL-17 and sputum neutrophil percentage, suggesting a potential role in the pathogenesis of severe early-onset phenotype.

## Introduction

Asthma is a heterogeneous disease of airways that is characterized by chronic airway inflammation, airway hyperactivity, reversible bronchoconstriction, and tissue remodeling [1]. In recent years, the prevalence of diagnosed asthma has been increasing in some countries. In particular, the control rate of asthma in China has been at a low level for a long time [2], which is becoming a threat to people's health. A latest epidemiological study showed that the asthma prevalence in Chinese adults is about 4.2%, while the diagnostic rate and inhaled hormone utilization rate in asthmatic care only 28.8% and 5.6% respectively [2]. The Global Initiative for Asthma (GINA) [1] pointed out that assessing the severity of asthma is a key step in the treatment of asthma. Dynamically monitoring the control level of asthma patients and identifying their predisposing factors can adjust the treatment plan as soon as possible to prevent further exacerbation. Although mild asthma is the most common form, about 8% of mild patients will develop into moderate to severe asthma [3]. Those with severe asthma have more frequent acute attacks, and the clinical symptoms and lung function deteriorate more seriously [1]. Therefore, early prevention and treatment of severe asthma are still major challenges facing physicians in China.

Age at asthma onset is now recognized as a critical factor in separating the heterogeneous phenotypes of asthma. A previous study has shown that late-onset asthma has fewer allergic reactions and severe conditions [4]. However, there has been very little research into the difference between severe early-onset and late-onset asthma, especially in relation to their inflammatory markers. Recent studies have shown that T helper (Th) 2-type cells, Th17 cells, airway smooth muscle (ASM) cells, and their cytokines play an important role in the pathogenesis of asthma [5]. Identifying the distinct inflammatory patterns, representing different phenotypes of severe asthma, can discriminate the underlying pathophysiological mechanisms, and therefore influence the strategies for asthma treatment. At present, the effects of some important inflammatory factors including herpes virus entry mediator (HVEM) [6], interleukin (IL)-5 [7, 8], and IL-17 [9] in the pathogenesis of asthma have attracted worldwide attention from scholars.

IL-17 is a pro-inflammatory cytokine produced by a subset of Th cells including Th17 cells and γδT cells. It has been demonstrated that IL-17 can activate airway neutrophils, induce neutrophil migration, and thus maintain the immune defense and inflammatory response in airway [9]. Several recent studies suggest an important role of IL-17 in regulating neutrophilic inflammation, airway hyperresponsiveness, and lung remodeling [10, 11]. However, there is no available information on the association between IL-17 and the development of early-onset asthma.

In this study, we aimed to compare the levels of inflammatory mediators between patients with severe early-onset and late-onset asthma, and determine the values of the markers in identifying specific phenotypes of severe asthma.

## Methods

### Study subjects

The present study consecutively enrolled 32 patients with severe early-onset asthma (age of onset < 12 years) and 32 patients with severe late-onset asthma (age of onset > 12 years) at Shenzhen People’s Hospital (the First Affiliated Hospital of Southern University of Science and Technology, the Second Clinical Medical College of Jinan University) from July 2018 to January 2019. All participants were aged > 18 years. According to the Global Initiative for Asthma (GINA) 2017 classification, all subjects met the diagnostic criteria of severe persistent asthma (therapy step 4 or 5). All asthma patients had a history of asthma, and preformed sputum induction, spirometry test, fractional exhaled nitric oxide (FeNO), and venous blood sampling. Based on the age of onset, all subjects were divided into 2 groups: early-onset group and late-onset group.

Patients who met the following criteria were excluded: (1) acute heart failure, severe organ failure, malignant tumors; (2) other lung diseases such as pulmonary infection, pulmonary hypertension, bronchiectasis which seriously affect lung function, sputum cell classification and other test results; (3) pregnancy and lactation; (4) recent surgery history; and (5) anybody has been accepted biologic therapies recently.

This research protocol was approved by the Medical Ethics Committee of Shenzhen People’s Hospital, and all subjects have signed written informed consent before enrolment in the study.

### Study design

On day 1 information of baseline characteristics and clinical scores of all subjects was collected, and the following determinations were also performed: FeNO, spirometry test, total Immunoglobulin E (IgE), antigen-specific IgE, IL-5, IL-17, HVEM, complete blood count (CBC), and differential leukocyte count (DLC). On day 3 induced sputum cell differential count from the subjects was detected.

### FeNO measurements

Before spirometry analyses, FeNO was measured by the NIOX MINO device (Aerocrine AB, Solna, Sweden)at an exhalation flow rate of 0.05 L/s for 10 s according to the manufacturer’s instructions. The FeNO measurements were consistent with the American Thoracic Society (ATS) / European Respiratory Society (ERS) 2005 guidelines methods [12], and the normal exhaled NO value was set as 5–35 part per billion (ppb) for healthy adults.

### Spirometry measurements

Spirometry test was performed using aV6200 spirometer (SensorMedics, USA) in accordance with the methods of the ATS/ERS task force to determine forced expiratory volume in 1 s (FEV1), FEV1 percentage of predicted (FEV1%pred), forced vital capacity (FVC) percentage of predicted (FVC%pred), FEV1/FVC ratio, maximal expiratory flow (MEF)25%, MEF50%, and maximal mid-expiratory flow (MMEF)75/25%. A predicted ratio for each parameter was calculated based on age, sex, height, and race.

### Total IgE and specific IgE measurements

Total serum IgE levels were determined by an electrochemiluminescence immunossay (ECLIA) method from Roche (Mannheim, Germany). Antigen-specific IgE was measured by the enzyme-linked immunosorbent assay (ELISA) kits (HOB Biotech Group, Suzhou, China), and allergen-specific IgE levels were considered positive sensitization by a value ≥ 0.35 IU/L.

### Serum cytokines and HVEM measurements

The serum levels of IL-5, IL-17 and HVEM were measured using ELISA kits according to the manufacturer’s instructions (Beiluo Biotechnology, Beijing, China).

### CBC and DLC tests

CBC and DLC parameters including red blood cells (RBCs), platelets and leukocytes were measured by an automated Sysmex XN-3000 (Sysmex Corporation, Kobe, Japan), using impedance and optic scatter method.

### Sputum induction and cell differential count

Sputum induction was performed with hypertonic saline inhalation, using a ultrasonic atomizer as previously described. The sample was considered qualified if it presented with cells viability ≥ 60% and ≤ 20% squamous cell. The cells were centrifuged and resuspended in saline, and sputum slides were prepared by cytocentrifugation for cytological examination. The slides were air-dried and stained with Giemsa. Differential cell count was measured by counting 400 nucleated cells per slide with high power (×400) magnification.

### Statistical analysis

SPSS 13.0 (SPSS Inc., Chicago, USA) was used for statistical evaluations. Normally parametric and nonparametric data are presented as means (standard deviation, SD) and medians (quartile 1 − quartile 3). The independent-samples t test was applied for difference in parametric variables between two groups, and the Mann–Whitney U test for the nonparametric data between two groups. Categorical variables were compared using the chi-square test. Correlation between the asthmatic risks and parameters were assessed by multivariate logistic regression analysis. Receiver operating characteristic (ROC) curves were generated to evaluate the values of biomarkers for distinguishing between two groups. Linear correlation analysis was performed to assess the linear relationship between two variables. A *P* value of less than 0.05 (*P* < 0.05) was considered as a statistically significant difference.

## Results

### Study population

The study population consisted of 64 adult subjects with severe asthma, with a mean age of 46.22 years (range 18–70 years), and of whom 53.1% were male. Their characteristics are shown in Table 1. In general, subjects with early-onset asthma were younger (40.53 ± 15.39 years vs. 51.91 ± 9.42 years, *P* = 0.001), had a longer duration of asthma (309.06 ± 199.28 months vs. 133.84 ± 121.87 months, *P* < 0.001), and were more likely to have a family history of asthma (53.1% vs. 25.0%, *P* = 0.021). Subjects with early-onset asthma also had higher MEF25% and MMEF75/25%, and were more sensitized to Dust mites (53.1% vs. 21.9%, *P* = 0.013) and Dermatophagoides farina (43.8% vs. 18.8%, *P* = 0.038).

**Table 1:** Comparison of demographic characteristics between early-onset group and late-onset group

	All (n = 64)	Early-onset group (n = 32)	Late-onset group (n = 32)	*χ*^2^/*t*	*P*
Sex					
Male, n (%)	34 (53.1)	18 (56.3)	16 (50)	0.251	0.616
Female, n (%)	30 (46.9)	14 (43.7)	16 (50)		
Age, (years), Mean ± SD	46.22 ± 13.90	40.53 ± 15.39	51.91 ± 9.42	3.566	0.001*
Age onset, (years), Mean ± SD	25.59 ± 17.87	9.78 ± 2.62	41.38 ± 11.26	15.46	0.000*
BMI, (kg/m^2^), Mean ± SD	23.97 ± 3.50	24.14 ± 3.35	23.82 ± 3.66	0.353	0.725
Length of hospital stay, (days), Mean ± SD	7.41 ± 2.57	7.75 ± 2.77	7.06 ± 2.34	1.072	0.288
Asthma duration, (months), Mean ± SD	221.45 ± 186.14	309.06 ± 199.28	133.84 ± 121.87	4.243	0.000*
Smoking history					
Yes, no quit, n (%)	9 (14.1)	3 (9.4)	6 (18.8)	1.956	0.376
Yes, quit, n (%)	10 (15.6)	4 (12.5)	6 (18.8)		
No, n (%)	45 (70.3)	25 (78.1)	20 (62.4)		
Hormone medication history, n (%)	19 (29.7)	13 (40.6)	6 (18.8)	3.668	0.055
Medication use, n (%)					
High dose ICS	54 (84.4)	25 (78.1)	29 (90.6)	1.896	0.168
Family history of asthma, n (%)	25 (39.1)	17 (53.1)	8 (25.0)	5.317	0.021*
ACT score, Median (Q1, Q3)	16.0 (14.0, 19.0)	16.0 (14.0, 18.0)	15.5 (14.0, 19.0)	0.061	0.951
Lung function					
FEV_1_, (L), Mean ± SD	2.03 ± 0.84	2.21 ± 1.04	1.87 ± 0.58	1.544	0.130
FEV_1_%pred, (%), Mean ± SD	68.43 ± 20.55	68.13 ± 23.69	68.70 ± 17.61	0.108	0.915
FVC%pred, (%), Mean ± SD	87.73 ± 17.29	84.43 ± 18.63	90.71 ± 15.67	1.429	0.158
FEV_1_/FVC, (%), Mean ± SD	63.87 ± 11.16	65.90 ± 13.37	62.03 ± 8.50	1.364	0.178
MEF50%, (%), Mean ± SD	39.05 ± 27.13	46.19 ± 34.87	32.58 ± 15.36	2.004	0.050
MEF25%, (%), Mean ± SD	29.69 ± 19.14	35.01 ± 25.21	24.86 ± 9.22	2.046	0.048*
MMEF75/25%, (%), Mean ± SD	34.78 ± 23.38	41.11 ± 30.35	29.04 ± 12.42	2.067	0.043*
Complications					
Sinusitis, n (%)	28 (43.8)	14 (43.8)	14 (43.8)	0.000	1.000
Allergic rhinitis, n (%)	22 (34.4)	13 (40.6)	9 (28.1)	1.108	0.292
Atopy, n (%)	37 (57.8)	21 (65.6)	16 (50.0)	1.602	0.206
Allergens					
Medicine, n (%)	14 (21.9)	9 (28.1)	5 (15.6)	1.463	0.226
Dust mites, n (%)	24 (37.5)	17 (53.1)	7 (21.9)	6.229	0.013*
Blattellagermanica, n (%)	22 (34.4)	13 (40.6)	9 (28.1)	0.931	0.335
*Aspergillus fumigatus*, n (%)	9 (14.1)	5 (15.6)	4 (12.5)	0.000	1.000
Shrimp, n (%)	17 (26.6)	10 (31.3)	7 (21.9)	0.601	0.438
Dermatophagoides farinae, n (%)	20 (31.3)	14 (43.8)	6 (18.8)	4.325	0.038*
Total IgE, (U/L), Median (Q1, Q3)	254.0 (86.39, 602.35)	319.7 (156.0, 607.8)	179.7 (69.74, 646.5)	0.969	0.332

SD: standard deviation; BMI: body mass index; ACT: asthma control test; Q1: quartile 1; Q3: quartile 3; ICS: Inhaled glucocorticoid; FEV_1_: forced expiratory volume in 1 s; FEV_1_%pred: FEV1 percentage of predicted; FVC: forced vital capacity; FVC%pred: FVC percentage of predicted; MEF: maximum expiratory flow; MMEF: maximal mid-expiratory flow

^*^Represent *P* < 0.05, suggest statistical significance

### Differential levels of inflammatory biomarkers between groups

**Fig. 1:**
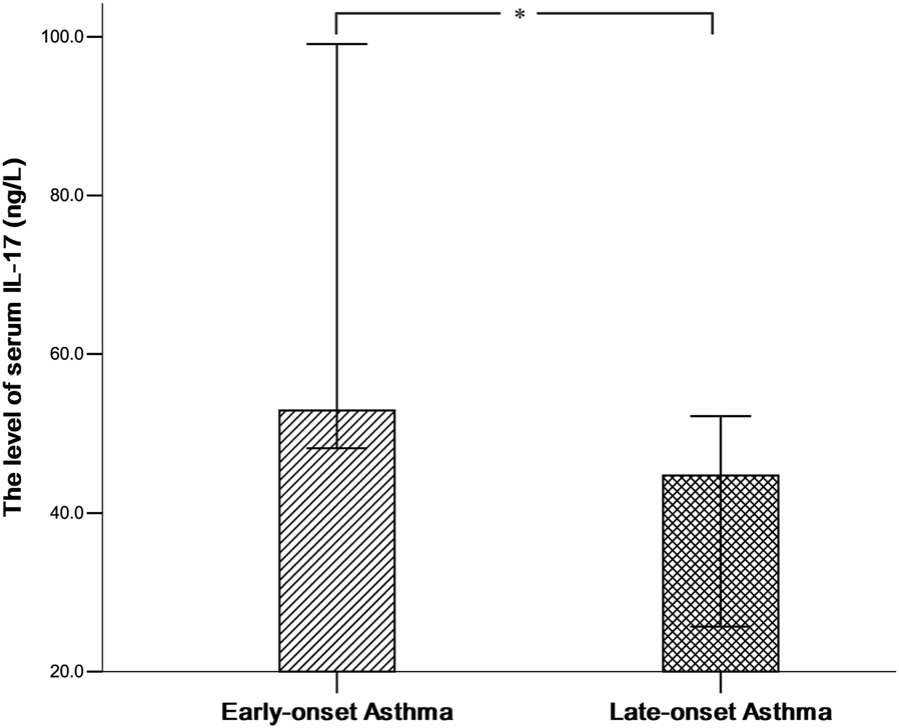
Differences of serum IL-17 levels between early-onset group and late-onset group

Compared to the patients with late-onset asthma, patients with early-onset asthma exhibited a higher median level of serum IL-17 [52.96(47.99, 105.50) vs. 44.75(20.81, 54.99) ng/L, *P* = 0.016] (shown in Fig. 1), and a higher neutrophils percentage in induced sputum samples (72.04 ± 26.90 vs. 53.61 ± 27.89%, *P* = 0.033). However, there were no significant differences in serum levels of IL-5 and HVEM between early-onset asthmatics and late-onset asthmatics. Other details are shown in Table 2.

**Table 2:** Comparison of laboratory indexes between early-onset group and late-onset group

	All (n = 64)	Early-onset group (n = 32)	Late-onset group (n = 32)	*t*/*Z*	*P*
Peripheral blood					
WBC, (10^9^/L), Mean ± SD	8.00 ± 2.29	8.25 ± 2.13	7.75 ± 2.46	0.860	0.393
Neu, (10^9^/L), Mean ± SD	5.30 ± 2.38	5.80 ± 2.17	5.02 ± 2.57	0.951	0.345
Eos%, (%), Median (Q1, Q3)	2.2 (0.2, 7.5)	1.0 (0.2, 7.3)	3.7 (0.2, 7.5)	1.717	0.241
Eos, (10^9^/L), Median (Q1, Q3)	0.22 (0.02, 0.53)	0.07 (0.01, 0.53)	0.28 (0.03, 0.53)	1.124	0.261
Neu/Eos, Median (Q1, Q3)	15.48 (6.79, 82.25)	24.92 (6.94, 131.91)	12.19 (6.05, 54.24)	0.737	0.461
IL-5, (ng/L), Median (Q1, Q3)	87.28 (35.79, 108.2)	89.54 (37.94, 104.34)	87.16 (29.49, 111.68)	0.156	0.876
IL-17, (ng/L), Median (Q1, Q3)	51.10 (40.25,81.98)	52.96 (47.99,105.50)	44.75 (20.81, 54.99)	2.400	0.016*
HVEM, (pg/L), Median (Q1, Q3)	87.23 (54.19, 109.2)	84.18 (57.21, 95.01)	94.75 (43.81, 115.69)	0.782	0.434
Induced sputum					
Neu%, (%), Mean ± SD	60.98 ± 28.68	72.04 ± 26.90	53.61 ± 27.89	2.202	0.033*
Eos%, (%), Median (Q1, Q3)	12.45 (1.40, 32.35)	3.8 (1.4, 14.4)	13.4 (2.8, 39.2)	1.490	0.136
Neu%/Eos%, Median (Q1, Q3)	5.99 (1.14, 48.40)	12.60 (3.23, 63.74)	2.62 (1.00, 23.25)	1.219	0.223
FeNO, (ppb), Median (Q1, Q3)	27.0 (16.0, 49.5)	22.5 (16.0, 47.3)	32.5 (16.0, 50.3)	0.559	0.576

WBC: white blood cell; SD: standard deviation; Neu: neutrophil; Eos: eosinophil; Q1: quartile 1; Q3: quartile 3; SB: standard bicarbonate; IL: interleukin; HVEM: herpes virus entry mediator; FeNO: fractional exhaled nitric oxide; ppb: part per billion

^*^Represent *P* < 0.05, suggest statistical significance

### Risk factors of severe early-onset phenotype of asthma

Multivariate logistic regression analysis showed that increased levels of serum IL-17 were tested as an independent risk factor for severe early-onset asthma [odds ratio (OR), 1.065; 95% confidence interval (CI), 1.012–1.121; *P* = 0.016] (shown in Table 3). However, neutrophils percentage of induced sputum was not associated with the severe early-onset phenotype (OR, 1.042; 95% CI 0.999–1.087; *P* > 0.05).

**Table 3:** Multivariable logistics regression analysis for severe early-onset asthma

	OR	95% CI	*P*
Serum IL-17	1.065	1.012 ~ 1.121	0.016*
Sputum Neu%	1.042	0.999 ~ 1.087	0.058

IL: interleukin; Neu: neutrophil; OR: odds ratio; CI: confidence interval

^*^Represent *P* < 0.05, suggest statistical significance

**Fig. 2:**
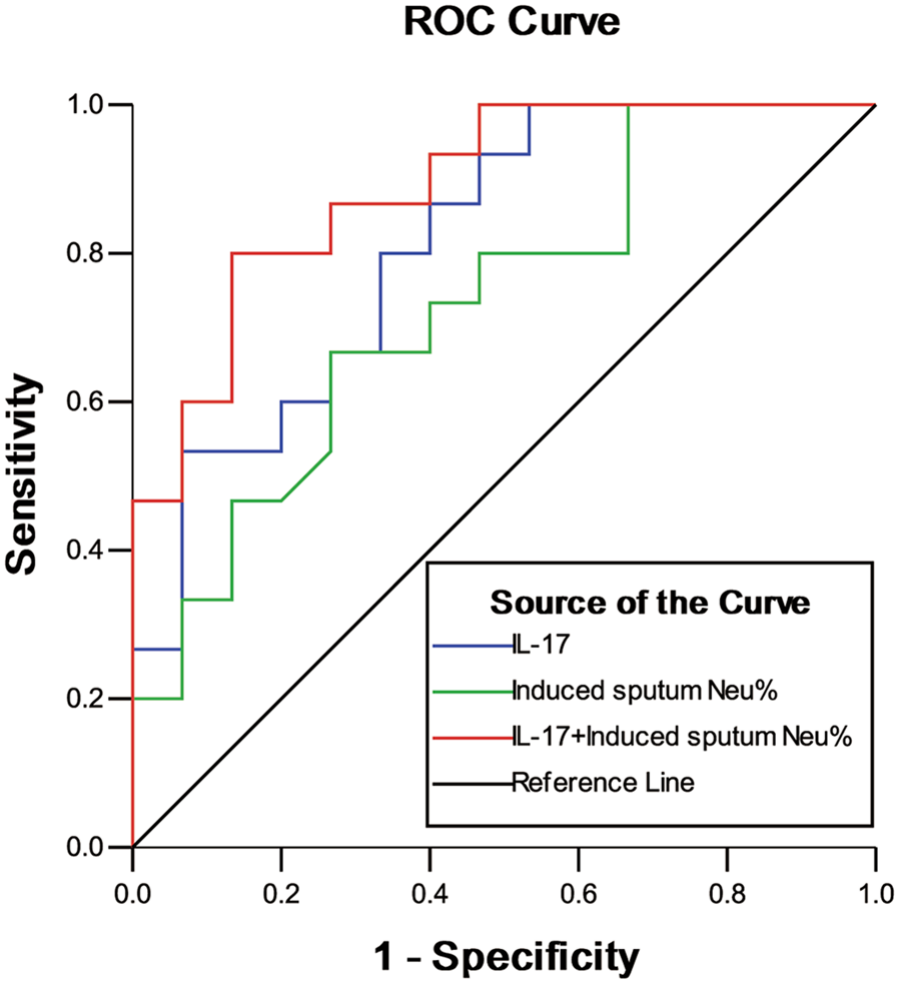
Receiver operating characteristic (ROC) curves for serum IL-17 and sputum neutrophils percent to differentiate between severe early-onset asthma and severe late-onset asthma

By performing the receiver operating characteristic (ROC) curve analysis, the area under the ROC curve (AUC) for serum IL-17 was highest (AUC = 0.813, *P* = 0.003), which had the optimal cut-off point of 35.22 ng/L with 100% sensitivity and 46.7% specificity. In contrast, the AUC of sputum neutrophils percentage was 0.731 (*P* = 0.031), and its optimal cut-off point was 66.10% with 66.7% sensitivity and 73.3% specificity. Moreover, serum IL-17 combined with sputum neutrophils percentage can increase the AUC to 0.889 (*P* < 0.001), with a sensitivity of 80.0% and a specificity of 86.7% (shown in Table 4 and Fig. 2).

**Table 4:** Differential diagnostic value of serum IL-17 and sputum neutrophils percent for severe early-onset asthma

	AUC	95% CI	Cut-off value	Sensitivity	Specificity	*P*
Serum IL-17	0.813	0.662–0.965	35.22	1.000	0.467	0.003*
Sputum Neu%	0.731	0.551–0.911	66.10	0.667	0.733	0.031*
Serum IL-17 + Sputum Neu%	0.889	0.775–1.003	–	0.800	0.867	0.000*

IL: interleukin; Neu: neutrophil; AUC: the area under the ROC curve; OR: odds ratio; CI: confidence interval

^*^Represent *P* < 0.05, suggest statistical significance

### Correlation between serum IL-17, sputum neutrophils, and spirometry parameters in severe early-onset phenotype of asthma

We next examined whether serum IL-17 and sputum neutrophils are correlated with spirometry parameters in patients with severe early-onset asthma. No correlations of significance were found between serum IL-17 or sputum neutrophils percentage and spirometry parameters (including FEV1%pred, FVC%pred, FEV1/FVC, MEF50%, MEF25%, and MMEF75/25%) in the early-onset group (*P* all > 0.05). Other details are shown in Table 5.

**Table 5:** Relationship between serum IL-17, sputum neutrophils percent, and Spirometry parameters in the early-onset group

	FEV_1_%pred		FVC%pred		FEV_1_/FVC		MEF50%		MEF25%		MMEF75/25%	
	r	*P*	r	*P*	r	*P*	r	*P*	r	*P*	r	*P*
Serum IL-17	0.270	0.279	0.149	0.556	0.317	0.200	0.181	0.473	0.260	0.298	0.223	0.374
Sputum Neu%	− 0.373	0.154	− 0.319	0.228	− 0.385	0.141	− 0.392	0.134	− 0.405	0.120	− 0.434	0.093

IL: interleukin; Neu: neutrophil; FEV_1_: forced expiratory volume in 1 s; FEV_1_%pred: FEV1 percentage of predicted; FVC: forced vital capacity; FVC%pred: FVC percentage of predicted; MEF: maximum expiratory flow; MMEF: maximal mid-expiratory flow

^*^Represent *P* < 0.05, suggest statistical significance

## Discussion

In the present study, we have shown the levels of serum IL-17 expression and sputum neutrophil percentage were increased in patients with severe early-onset asthma compared with those in patients with severe late-onset asthma, but no difference was observed in other inflammatory biomarkers between the two groups. It has been hypothesized that neutrophils and the cytokine IL-17 play a potential role in severe asthmatics. This may explain why elevated levels of sputum neutrophilia and serum IL-17 were found in subjects with severe early-onset asthma, although early-onset asthma was customarily linked to eosinophilic inflammation and atopic status.

Our study assessed 64 adult patients admitted with acute severe asthma (step 4 or 5), and 50.0% of them presented asthma onset before 12 years of age. The two groups had familiar smoking rate (early-onset vs. late-onset: 27.9% vs. 37.5%), and presented familiar prevalence of atopy (early-onset vs. late-onset: 65.6% vs. 50.0%). Furthermore, the allergy blood tests showed that the patients of both groups shared many features of allergic conditions, and consistent with previous researches [13, 14], patients with early-onset asthma were more prone to be allergic to dust mite and dermatophagoides farina. However, we found that the patients in the early-onset group had a higher family history of asthma (*P* = 0.021) and were significantly younger (*P* = 0.001) in comparison with those in the late-onset group. It suggested that patients with early-onset asthma may be more likely to develop severe asthma with a family history and at a younger age.

In both groups of early and adult onset asthma, patients with severe asthma were characterized by decreased FEV1% of the predicted value (68.43 ± 20.55%) and FEV1/FVC% (63.87 ± 11.16%). Nevertheless, the levels of MEF25% and MEF75/25% in the early-onset asthma group were significantly higher than those in the late-onset asthma group (*P* = 0.048 and 0.043, respectively). It suggested that although early-onset asthmatics have a longer disease duration, the level and rate of decline in lung function are not greater than those of late-onset asthmatics. Moreover, the function of small airway is significantly better than that of patients with late-onset asthma, and this finding was compatible with the literature [13]. Whereas other researchers hold the opinion that late-onset asthmatics are more likely to have persistent airway airflow limitation, and overreliance on short-acting β2-agonists and inhaled corticosteroid [15, 16]. Different definitions and study populations may count for the discrepancies. Lung function in adulthood declines with age and disease duration, and increased incidence of persistent airflow limitation may be observed in patients with severe asthma [2]. Hence, we should closely monitor the lung function of patients with early-onset asthma in adults, and intervene promptly to prevent further deterioration of lung function.

In the present study, sputum neutrophil percentage was elevated in the early-onset group compared to the late-onset group (72.04 ± 26.90% vs. 53.61 ± 27.89%), and there was a higher level of serum IL-17 in the early-onset group [52.96 (47.99, 105.50) ng/L for early-onset vs. 44.75 (20.81, 54.99) ng/L for late-onset]. Furthermore, the multivariate logistic regression analysis revealed that IL-17 was an independent risk factor for early-onset severe asthma. Although early-onset asthma phenotype was associated with eosinophilic airway inflammation as previously described, no significant differences in FeNO levels, blood eosinophils and sputum eosinophils were observed between the two groups in our study. These results may have significant clinical implications. IL-17 has been shown to have a major role in the recruitment and activation of neutrophils. Th-17 regulatory cytokines, IL-21 and IL-23, can stimulate neutrophils infiltrating in the lung tissue to produce IL-17 via STAT3 signaling pathway [17], and high levels of IL-17 would trigger high production of reactive oxygen radicals (ROS) [18], which contributed to inflammatory response exacerbation and airway remodeling. If IL-17-induced neutrophilic airway inflammation is proven to be involved in the pathogenesis of severe early-onset asthma, it could lead to a new therapeutic option.

While raised serum levels of IL-17 has been shown to correlate with bronchial hyper-responsiveness and airway remodeling [10], its role as a diagnostic assessment for specific phenotype of severe asthma is not clear. The results of ROC curve analysis showed that serum IL-17 had an AUC value of 0.813, and the sensitivity and specificity were 100% and 46.7%, respectively, at the best cut-off value. Thus, serum IL-17 can be used to identify the early-onset phenotype among the patients with severe asthma. In order to improve the diagnostic efficacy, we combined of serum IL-17 and sputum neutrophil percentage. The diagnostic tests showed the combination of two biomarkers achieved the best discriminatory power, with AUC of 0.889, and provided a reasonable sensitivity of 80.0% and specificity of 86.7%. This finding suggests that patients with severe early-onset asthma may be more likely to have IL-17-mediated neutrophilic inflammation compared to those with severe late-onset asthma. However, no relationship was found between serum IL-17, sputum neutrophil percentage, and lung function parameters.

This study has some limitations. First, this is a single-center study with small sample size, and therefore the generalization of the findings is limited. Second, onset age of asthma could have been mistaken. To minimize such recall bias and misclassification, we selected the cut-off age of 12 years which is consistent with memorable personal histories. Third, smoking is a potential variable in inducing immunologic changes, but due to the small sample size, we did not include smoking in the exclusion criteria. However, we compared the smoking status between the two groups, and no statistical difference was found. Finally, we did not collect the data for patients with mild or moderate asthma, hence the associations between the biomarkers and the severity of early-onset asthma could not be evaluated.

In general, both severe early-onset and late-onset asthma appear to have similar decline in lung function despite different disease durations. Furthermore, higher levels of serum IL-17 and sputum neutrophil were found in severe early-onset asthma compared with severe late-onset asthma. These findings highlight the potential role of IL-17-mediated neutrophilic inflammation in the pathogenesis of severe early-onset asthma, thus suggesting a new therapeutic option for targeting the IL-17 pathway.
